# Optimization of decision thresholds for Mycobacterium tuberculosis can effectively improve the performance of mNGS in tuberculosis diagnosis

**DOI:** 10.3389/fcimb.2025.1646194

**Published:** 2025-09-11

**Authors:** Yuecui Li, Lili Zhang, Guannan Ma, Chenghang Li, Weiyue Hu, Ruotong Ren, Yinghui Zang, Dandan Ying, Shuai Qiu, Shuyan Jin, Chunjie Qiu, Xuefang Cao

**Affiliations:** ^1^ Department of Infectious Disease, The First People’s Hospital of Yongkang, Affiliated to Hangzhou Medical College, Jinhua, China; ^2^ Zhejiang Key Laboratory of Digital Technology in Medical Diagnostics, Hangzhou, China; ^3^ MatriDx Biotechnology Co., Ltd, Hangzhou, China

**Keywords:** tuberculosis, bronchoalveolar lavage fluid, metagenomic next-generation sequencing, threshold optimization, diagnostic efficacy

## Abstract

**Background:**

Pulmonary tuberculosis (TB) diagnosis remains challenging due to limitations in traditional methods. This study aimed to optimize the metagenomic next-generation sequencing (mNGS) threshold for Mycobacterium tuberculosis complex (MTBC) detection and evaluate its efficacy compared to standard diagnostic approaches.

**Methods:**

A total of 264 bronchoalveolar lavage fluid (BALF) samples were collected from patients with suspected pulmonary TB at Yongkang First People’s Hospital between January 2022 and June 2023. After excluding patients with incomplete data, 59 clinically confirmed TB patients and 111 with non-tuberculous conditions were enrolled. mNGS data were analyzed to calculate reads per million (RPM) for MTBC, and thresholds of 0.02, 0.05, and 0.10 RPM were evaluated for diagnostic efficacy using clinical diagnosis as the gold standard.

**Results:**

The area under the receiver operating characteristic (ROC) curve (AUC) for mNGS in diagnosing TB at RPM thresholds of ≥0.02, ≥0.05, and ≥0.10 were 0.881, 0.873, and 0.814, respectively. The optimal detection threshold was found at RPM ≥ 0.05. Comparative analysis showed mNGS (AUC = 0.873) outperformed routine culture (0.718), PCR (0.741), and Xpert (0.763). Combining mNGS with these methods improved AUC values to 0.837, 0.868, and 0.873, respectively.

**Conclusion:**

Optimizing the mNGS threshold to ≥0.05 significantly enhances MTBC detection in pulmonary TB. Combining mNGS with traditional methods further improves diagnostic efficacy, suggesting a potential role for mNGS in clinical TB management.

## Introduction

Tuberculosis (TB), caused by the Mycobacterium tuberculosis complex (MTBC), remains a significant global public health challenge, characterized by high morbidity and mortality rates in humans (Huang et al., 2022). Although the incidence of tuberculosis has gradually declined over the past decade, with mortality rates dropping by nearly one-third, this positive trend was abruptly disrupted by the COVID-19 pandemic. In many regions, the pandemic led to a substantial reduction in tuberculosis testing and case notifications, contributing to an increase in mortality and reversing a decade of progress in global tuberculosis control (Dheda et al., 2022). It is estimated that 10.6 million people will be infected with tuberculosis globally in 2021, equivalent to 134 cases of tuberculosis per 100,000 people. The diagnosis and treatment of tuberculosis in clinical practice presents significant challenges and controversies (Ding et al., 2021). Numerous studies have revealed co-infection with various strains of Mycobacterium or other pathogenic species in patients with pulmonary tuberculosis (Huang et al., 2010; Shi et al., 2020). Given that rapid and accurate diagnosis is essential for effective treatment of tuberculosis, there is an urgent need for the development and implementation of rapid screening and diagnostic methods to enhance the management of TB.

Recently, metagenomic next-generation sequencing (mNGS) has rapidly developed as a complementary method widely used for diagnosing various infectious diseases in clinical setting. With its Shorter turnaround time, unbiased detection, and semi-quantitative value, mNGS can theoretically identify all pathogens present in a clinical sample, making it particularly suitable for diagnosing rare, novel, and atypical etiologies of complicated infectious diseases. This capability enables more precise guidance for targeted antimicrobial therapy (Chiu and Miller, 2019; Han et al., 2019). Extensive studies have demonstrated that mNGS is becoming an important complement to etiologic diagnostic workflows for TB patients due to its superior performance (Sun et al., 2021; Liu et al., 2022). However, significant challenges persist in assessing the efficacy of mNGS for MTBC detection. Variability in the genomic copy number of MTBC in patient samples, coupled with difference in mNGS methodologies and data analysis approaches employed exclusively in previous studies, complicates this assessment. Many studies have either focused on unique MTBC reads instead of employing a semi-quantitative metric, such as Specific reads per million reads (RPM), as a fundamental parameter or failed to define a specific RPM threshold for MTBC detection. As a result, these factors hinder the development of a comparable semi-quantitative assay and obstruct validated investigations regarding the efficacy of mNGS for TB evaluation in a large cohort.

In this study, we conducted a retrospective cohort study to analyze and determine the optimal RPM detection threshold of mNGS for the detection of MTBC using mNGS data from bronchoalveolar lavage fluid (BALF) samples of patients with clinically diagnosed TB and non-TB disease. Furthermore, the adjunctive diagnostic efficacy of threshold-optimized mNGS for MTBC was comprehensively evaluated by comparing it with clinically routine MTBC detection methods including culture, PCR and X-pert.

## Methods

### Study design and patients

The present study retrospectively enrolled patients with suspected pulmonary tuberculosis from January 2022 to June 2023 at Yongkang First People’s Hospital. Demographic information. Clinical symptoms, laboratory test results, imaging examination results, diagnosis and treatment history, and outcomes were collected from electronic medical records. Patients excluded were those who have incomplete clinical data, or those who without anyone of microbial results, such as culture, PCR or X-pert. Suspected tuberculosis (TB) included met any of the following criteria: 1) Symptoms of TB poisoning such as cough, fever, night sweats or weight loss or 2) Imaging features of tuberculosis.

### Definitions of TB cases

The final clinical diagnostic criteria for pulmonary tuberculosis cases was positive for microbial confirmed. For patients without any microbial evidence, the physician has diagnosed tuberculosis by combining the imaging and clinical manifestations of the patients, with excluding other diseases and histopathological confirmed TB, together with the patient’s confirmed responsiveness to anti-TB treatment after one month of follow-up.

Clinicians ultimately diagnosed TB cases according to the clinical guidelines of the Ministry of Health of the People’s Republic of China. Defined TB cases should comply with any of the following criteria: (1) The microbiological results (including acid-fast staining smear and culture, X-pert, PCR, mNGS) were positive. (2) The pathological result of lung biopsy tissue is consistent with the pathological features of tuberculosis. (3) The lung lesions reduced or disappeared after three months of antituberculosis treatment. Otherwise, they are classified as non-TB cases.

Clinical confirmed pulmonary TB was defined as a patient who presented with typical chest imaging suggestive of TB and met the criteria (typical symptoms of pulmonary TB, positive interferon-gamma release assay (IGRA), or positive purified protein derivative (PPD) test (or tuberculin skin test (TST)), positive culture results, nucleic acid test or acid-fast bacilli (AFB) smear for Mycobacterium tuberculosis (MTB) detection in sputum or BALF.

The study was approved by the Ethics Committee of Yongkang First People’s Hospital and all data were anonymized prior to analysis. The study was conducted in accordance with the Declaration of Helsinki and the study data were obtained from Department of Infectious Diseases, Yongkang First People’s Hospital. Informed consent was obtained from all participants or their legal guardians.

### Specimen collection and laboratory procedures

BALF samples were collected by experienced bronchoscopists through bronchoscopy based on canonical operational procedures (Meyer et al., 2012). Saline (60 mL~100 mL) was injected into the bronchial lumen of the segment and withdrawn after washing briefly. The qualified BALF (at least 25 mL) was divided into 5 parts, 4 of which were subjected to traditional test (AFB staining and culture), MTB PCR, and X-pert MTB/RIF assay, and the rest were stored in a sterile container at -20 °C, and sent to MatriDx Biotechnology for mNGS testing. BALF samples for PCR and X-pert MTB/RIF assay were processed following the manufacturer’s instructions.

### mNGS procedure and analysis

mNGS workflow comprised four sequential steps: sample processing, nucleic acid extraction, library preparation, and bioinformatics analysis. To maximize inter-laboratory reproducibility, all wet-lab and in-silico procedures followed a locked Standard Operating Procedure (SOP) with explicit QC metrics. Basically, according to the manufacturer’s instructions, DNA was extracted from the supernatant obtained after tissue pretreatment using a magnetic bead-based method (Genskey Co., Ltd., China). Following quantification and normalization of DNA concentration, a DNA library was prepared following the protocol of the NEBNext Ultra II DNA Library Prep Kit for Illumina (New England Biolabs Inc.). This process included enzymatic fragmentation, end repair, barcode ligation, library purification, and PCR amplification. The resulting DNA library quality was evaluated using the Qubit dsDNA High Sensitivity (HS) Assay Kit (Thermo Fisher Scientific, USA). Libraries that met the quality criteria were sequenced on the MGISEQ-200RS platform (MGI, China) using the MGISEQ-200RS High-throughput Sequencing Reagent Kit (FCL SE50). To ensure data quality, raw sequencing reads were filtered using the fastp software to remove low-quality reads, adaptor contamination, duplicates, and reads shorter than 50 base pairs. Reads aligning to the human reference genome (hg38) were excluded. The remaining reads were normalized to 20 million per sample and subjected to multi-sequence alignment. Microbial identification was performed by aligning the filtered reads against an in-house pathogenic microorganism genome database. It’s worth noting that, before data analysis, microbes detected in clinical samples were first compared with those detected in the parallel NTC (no template control). Mycobacterium tuberculosis was considered positive when at least 1 read is mapped to MTBC (strictly mapped to the number of sequences at the genus level).

### Statistical analysis

All collected data were statistically analyzed using R packages. Cutoff value for TB diagnosis was evaluated by receiver operating characteristic (ROC) curves. The area under curve (AUC), accuracy (ACC), sensitivity (SEN), specificity (SPE), positive predictive value (PPV), negative predictive value (NPV), percentage of positive accordance (PPA) and percentage of negative accordance (NPA) were calculated for routine test, PCR, X-pert, mNGS and their combinations using clinical diagnosis as the reference standard. The comparison of ROC curves was performed using the method of Delong. *P*-value < 0.05 was considered statistically significant in this study. Pairwise comparisons among indicators (including SEN, SPE, PPA, ACC, PPV, NPV and NPA) were conducted using the McNemar’s exact test for paired data. All tests were two-sided, and the significance level was set at P < 0.05.

We conducted a decision curve analysis (DCA) to assess the clinical utility of the mNGS threshold (RPM ≥0.05) for diagnosing tuberculosis (TB). The analysis was based on a dataset of 170 BALF samples, including 59 confirmed TB cases and 111 non-TB cases. We calibrated the raw RPM values using logistic regression to convert them into continuous probabilities (ranging from 0 to 1). We evaluated the net benefit of using the mNGS probability across a range of threshold probabilities (0% to 60%), comparing it to two strategies: “Treat All” (treat all patients) and “Treat None” (treat no patients). The RPM ≥0.05 threshold was specifically marked on the decision curve to visualize its position relative to the net benefit and extreme strategies.

## Results

### Optimization of mNGS threshold for MTBC detection

**Figure 1:**
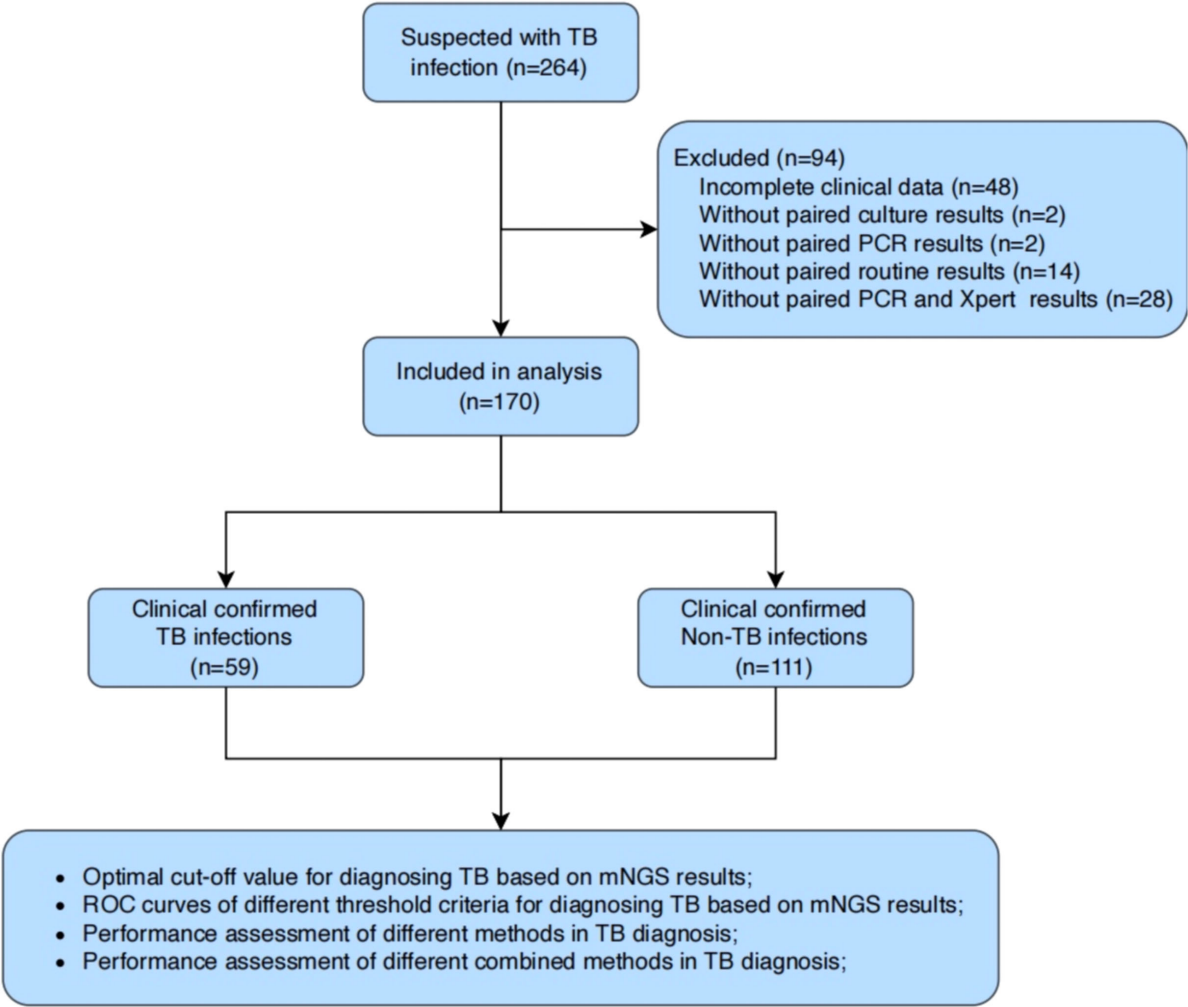
Flowchart of patients enrolled in this study.

A total of 264 BALF samples were collected from patients with suspected pulmonary tuberculosis at Yongkang First People’s Hospital from January 2022 to June 2023 (**Figure 1**). After excluding patients with incomplete clinical data or lacking microbiological results (e.g., culture, PCR, or X-pert), we enrolled 59 patients with clinically confirmed tuberculosis (TB, n=59) and 111 patients with clinically confirmed non-tuberculous tuberculosis (Non-TB, n=111) were finally enrolled. All BALF samples from these 170 patients were subjected to mNGS.

**Figure 2:**
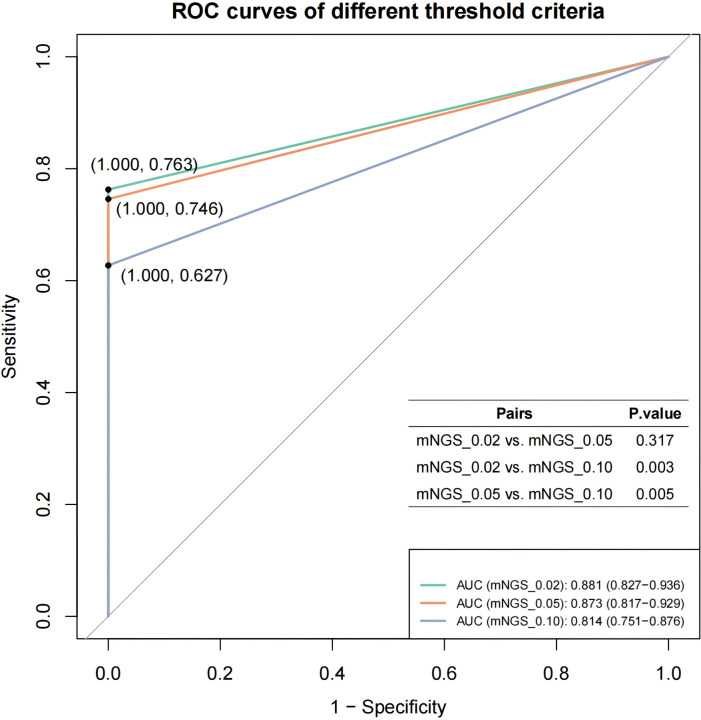
The ROC curves of different threshold criteria.

The mNGS-detected MTBC reads were normalized using the total mNGS data volume of the corresponding samples to obtain Specific reads per million reads (RPM) as the semi-quantitative baseline data for MTBC. Based on the distribution characteristics of RPM values in MTBC-positive samples, we selected thresholds of 0.02, 0.05 and 0.10 RPM for optimization. We pooled the mNGS data from MTBC-positive samples according to the aggregated mNGS dataset, and evaluated the diagnostic efficacy of mNGS for tuberculosis at each threshold, using clinical diagnosis of TB/non-TB as the gold standard. The results demonstrated that the AUC values for mNGS in adjunctive diagnosis of TB at the RPM thresholds of ≥0.02, ≥0.05 and ≥0.10 were 0.881, 0.873 and 0.814, respectively (**Figure 2**). Furthermore, t-test analysis indicated that the AUC values for RPM≥0.02 (*P* = 0.003) and RPM≥0.05 (*P* = 0.005) were significantly higher than that of RPM≥0.10 (**Figure 2**). This suggests that the optimal threshold for mNGS detection of MTBC should be set at 0.05 or lower. Notably, the AUC values for RPM≥0.02 and RPM≥0.05 were not significantly different (*P* = 0.317) (**Figure 2**). Considering the challenges associated with detecting the MTBC genome as an intracellular bacterium and the inherent sensitivity limitations of mNGS, RPM≥0.05 was finally selected as the threshold value in the subsequent comparative study on the performance of multi-methods in this study.

### Efficacy evaluation of the threshold-optimized mNGS in MTBC detection

**Figure 3:**
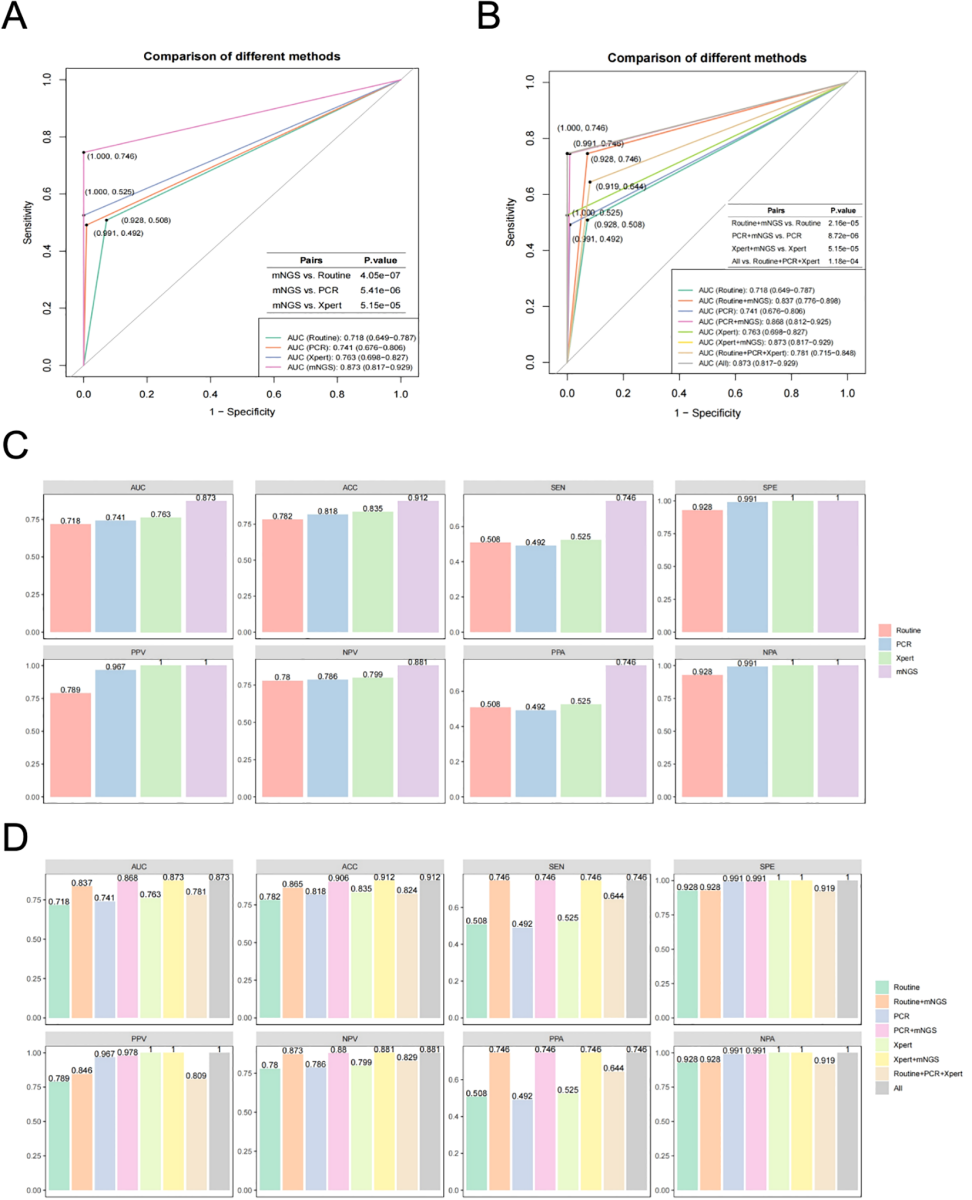
Efficacy evaluation of the threshold-optimised mNGS for MTBC detection. **(A)** ROC curves for mNGS compared to various traditional methods (routine culture, PCR and X-pert). **(B)** ROC curves for combined approaches (mNGS+routine culture, mNGS+PCR and mNGS+X-pert) compared to individual traditional methods and overall performace (All). **(C)** Comparison of area under curve (AUC), accuracy (ACC), sensitivity (SEN), specificity (SPE), positive predictive value (PPV), negative predictive value (NPV), percentage of positive accordance (PPA) and percentage of negative accordance (NPA) between mNGS and traditional methods. **(D)** Comparison of AUC, ACC, SEN, SPE, PPV, NPV, PPA and NPA between combined approaches (mNGS+Routine culture, mNGS+PCR and mNGS+X-pert), traditional methods and overall performance.

As described above, the positive determination of MTBC detected by mNGS was initially performed using the threshold of RPM≥0.05. Samples were classified into MTBC positive and negative group, and corresponding samples were pooled for further analysis based on results obtained from routine culture, PCR and X-pert methods. Ultimately, we evaluated the methodological efficacy of these different diagnostic approaches and their combinations for the adjunctive diagnosis of MTBC, using clinical diagnoses of TB or non-TB as the gold standard. The comparative analysis of the efficacy of multiple methods for TB diagnosis, based on ROC curves, revealed that the AUC values of mNGS, routine culture, PCR and X-pert were 0.873, 0.718, 0.741 and 0.763, respectively (**Figure 3A**). The AUC for mNGS was significantly higher than those of routine culture, PCR and X-pert, with *P*-values of 4.05e-07, 5.41e-06 and 5.15e-05, respectively (**Figure 3A**). These findings suggest that threshold-optimized mNGS demonstrates superior efficacy in detecting MTBC compared to the routinely used clinical assays (routine culture, PCR and X-pert).

Furthermore, we explored the combination of threshold-optimized mNGS with routinely used clinical MTBC assays. The results showed that when combined with routine culture (AUC = 0.718), PCR (AUC = 0.741), and X-pert (AUC = 0.763) individually, the AUC values for the combined approaches, mNGS+Routine culture, mNGS+PCR and mNGS+X-pert, were significantly higher, with AUC values of 0.837, 0.868 and 0.873, respectively (**Figure 3B**). The *P*-values for these comparisons were all less than 0.05 (2.16e-05, 8.72e-06 and 5.15e-05, respectively) (**Figure 3B**). The diagnostic strategy using mNGS+Xpert, the number of false-negative cases decreased from 28 to 15, indicating 13 additional cases were correctly identified, reflecting improved diagnostic sensitivity. The net correct reclassification was 13 cases, corresponding to a Net Reclassification Improvement (NRI) of 0.08%, with a Number Needed to Test (NNT) of 13.1—meaning one additional true case would be identified for every 13 individuals tested (**Supplementary Table S1**). These results indicate that combining threshold-optimized mNGS with standard clinical assays improves the diagnostic efficacy for detecting MTBC.

In addition to the AUC, we assessed the efficacy of various MTBC assays using ROC curves based on additional parameters related to diagnostic performance, including ACC, SEN, SPE, PPV, NPV, PPA and NPV (**Figures 3C, D**). The results showed that the threshold-optimized mNGS demonstrated superior performance compared to routinely used clinical MTBC assays (routine culture, PCR and X-pert), with the highest values for ACC (0.912), SEN (0.746), SPE (1.000), PPV (1.000), NPV (0.881), PPA (0.746) and NPA (1.000). Notably, four parameters (ACC, SEN, NPV and PPA) showed significant improvements relative to other assays (**Figure 3C**). Additionally, when compared to routine culture, PCR and X-pert alone, the various combinations of assays, including with mNGS with routine culture, mNGS with PCR, and mNGS with X-pert, also exhibited significant enhancements in AUC, ACC, SEN, NPV and PPA (**Figure 3D**).

The DCA curve shows that the orange curve (RPM ≥ 0.05) outperforms the other strategies (Treatment All and Treatment None) between approximately 5% and 35% threshold probabilities, indicating the highest clinical net benefit in this range. The red dashed line marks the RPM ≥ 0.05 threshold, which falls within this optimal net benefit region, confirming its validity. Below 5%, the orange curve aligns closely with the Treatment All strategy, suggesting a high false positive rate in this range. Above 35%, the orange curve maintains a higher net benefit than Treatment None, showing that RPM ≥ 0.05 is still more beneficial than no treatment. The orange curve remains stable and above the Treatment None line beyond 40%, indicating consistent clinical value even at higher threshold probabilities (**Supplementary Figure S1**).

## Discussion

TB is a serious threat to human health, so precise diagnosis and treatment of TB has attracted much attention. Common diagnostic methods for TB include tuberculin skin test, TSPOT.TB assay, acid-resistant staining, mycobacterial culture, and molecular diagnostic techniques such as PCR and X-pert MTB/RIF (Acharya et al., 2020). However, challenges associated with culture techniques and the limitations in sensitivity and specificity of conventional methods often result in these tests failing to meet clinical needs in a timely manner. Therefore, there is an urgent need for advanced precision diagnostic methods and *in vitro* diagnostic products for TB that are rapid, convenient, accurate, and cost-effective.

As we know, mNGS is regarded as an important complementary diagnostic method for identifying pathogenic microorganisms (Gu et al., 2019) This approach enables the rapid screening of difficult-to-cultivate or rare microorganisms, as well as the prompt identification of known pathogens such as TB, and detection of drug resistance genes (Gautam et al., 2018; Guthrie et al., 2018; Borroni et al., 2019; Chiu and Miller, 2019). Indeed, mNGS has been widely utilized for the diagnosis of pathogenic microorganisms and for guiding clinical treatment in various infections, including central nervous system infections, lung infections, bloodstream infections, ocular diseases, and infections in immunocompromised patients. Several clinical application studies that have demonstrated that mNGS enhances the clinical detection of MTBC and provides adjunctive diagnostic value for TB compared to conventional diagnostic methods (Liu et al., 2021; Liu et al., 2022). Despite rapid advancements in metagenomic next-generation sequencing (mNGS) and its growing application in clinical diagnostics, several major challenges remain in data analysis and clinical interpretation. These include the absence of standardized workflows and quality control measures, interference from high host DNA background and limited microbial biomass, difficulties in interpreting large volumes of complex data, limitations in current databases and bioinformatics pipelines, and the need for effective integration of sequencing results with clinical context (Simner et al., 2018; Laudadio et al., 2019; Liu and Ma, 2024; Zhao et al., 2024). Key questions include how to ensure comparability of mNGS data for the same pathogenic microorganisms across different samples and how to establish an appropriate threshold for positive determination of mNGS results for identical pathogens. These issues present substantial challenges for clinicians interpreting mNGS data in patients with suspected TB and appear to have been inadequately addressed in existing studies.

To address the challenges associated with mNGS in detecting MTBC, this study analyzed the RPM of MTBC from patients’ BALF samples and its correlation with clinical TB diagnosis as gold standard. A retrospective cohort study was conducted comprising 59 patients clinically diagnosed with TB and 111 patients diagnosed with non-TB. Our study demonstrated a high diagnostic performance of mNGS for pulmonary TB using BALF specimens (AUC = 0.873), which is consistent with previous findings, such as study (Liu et al., 2021). This similarity across independent cohorts supports the reproducibility and generalizability of mNGS in diverse clinical settings. Both studies employed mNGS in TB-suspected patients using BALF samples, suggesting a stable diagnostic capability of this technique. While there are differences in study design-including sample size [170 in our study *vs*. 322 in (Liu et al., 2021)], reference standards, and patient inclusion criteria—the convergence of diagnostic accuracy suggests that mNGS offers robust performance irrespective of these contextual variations. Additionally, we also compared the performance of mNGS at the optimal threshold (MTBC RPM≥0.05) with that of conventional MTBC detection methods, including culture, PCR, and Xpert. The results suggest that optimizing the threshold can enhance the interpretability of mNGS data, enabling clinicians to utilize this information more confidently for efficient diagnosis of MTBC. The decision curve analysis showed that our model provided a higher net benefit compared to the “treat all” or “treat none” strategies across a range of clinically relevant threshold probabilities (0.1 to 0.6), indicating its practical value in guiding clinical decision-making (**Supplementary Figure S2**).

Furthermore, we also found that the combination of mNGS with routine culture, mNGS with PCR, and mNGS with X-pert exhibited significantly higher efficacy in determining TB than any of the conventional methods alone. This finding aligns with previous studies (Zhou et al., 2019), while our work provides more concrete data to support this conclusion.

### Clinical practice and challenges

In translating the ≥0.05 RPM threshold into routine process, three real barriers must be weighed.

- Turnaround time (TAT): Our in-house workflow delivers results within 24–48 h (median 30 h), exceeding the 2 h of Xpert MTB/RIF. Shortening TAT to <24 h is feasible with same-day batching and rapid library prep kits (e.g., Illumina DNA Prep Mx), but requires 24-hour sequencing core staffing. We therefore propose tiered algorithms: smear/Xpert for initial triage, reserving mNGS for smear-negative, clinically suspicious cases where delayed TAT is acceptable.
- Cost and infrastructure: Reagent costs are currently ≈US$250 per mNGS test (library prep + sequencing) versus ≈US$10 for smear microscopy and ≈US$15–20 for Xpert (cartridge-based). However, the higher specificity (100%) of the 0.05 RPM threshold has the opportunity to offsets downstream costs by averting empiric multi-drug therapy in false-positive patients. In low-resource settings lacking NGS infrastructure, regional sequencing centers receiving couriered samples or cloud-based bioinformatics (upload FASTQ to secure servers) also can reduce capital outlay.
- Balancing sensitivity and specificity to prevent overtreatment: The selected 0.05 RPM threshold yields sensitivity 74.6% and specificity 100% in our cohort. The zero false-positive rate at this cut-off minimizes the risk of unnecessary anti-TB therapy and its attendant toxicities, a critical advantage where second-line drug resources are scarce.

### Limitations

While our study establishes a preliminary RPM threshold (≥0.05) for MTBC detection by mNGS, several limitations must be acknowledged. First, the retrospective, single-center design and moderate sample size (n = 170) restrict the generalizability of the threshold to populations with distinct MTBC strain diversity, HIV prevalence, or pediatric paucibacillary disease. Additionally, the sample composition may not fully represent patients with rare co-infections or atypical presentations, requiring further validation in larger, multi-center cohorts.

Second, the threshold’s applicability to other sample types, such as sputum or cerebrospinal fluid (CSF), remains uncertain. Different sample types may exhibit varying pathogen loads and levels of host DNA contamination, which could affect the optimal RPM threshold. For instance, sputum samples may have a higher bacterial load, while CSF samples might have lower pathogen concentrations. Therefore, further studies are necessary to determine if this threshold holds for other sample types commonly used in clinical practice.

Third, inter-laboratory reproducibility remains unexplored; variations in DNA extraction kits, sequencing depth (< 20M reads), or bioinformatic pipelines (e.g., k-mer *vs*. alignment-based) could necessitate recalibration of the threshold across different laboratories and sequencing platforms. Different sequencing platforms may yield varying results due to differences in depth, error rates, and sensitivity, making the threshold potentially platform-dependent.

We also did not evaluate the threshold in immunocompromised hosts (e.g., transplant recipients), where atypical bacterial loads and co-infections could alter RPM distributions. The absence of longitudinal data precludes assessment of threshold stability during anti-TB therapy or across specimen types (BALF *vs*. sputum *vs*. CSF). Finally, real-world implementation must consider infrastructure, turnaround time, cost-effectiveness, and regulatory validation—none of which were formally assessed here. Multicenter, prospective studies incorporating health-economic modeling in resource-constrained environments are therefore warranted.

## Conclusions

In summary, the results of the present study suggest that optimizing thresholds for mNGS may enhance the efficiency of clinical detection of MTBC and improve the accurate diagnosis of TB. This optimization could lead to additional patient benefits, including increased diagnostic efficacy, timeliness, and reduced clinical costs. Ultimately, the implementation of such threshold adjustments provides a more efficient, convenient, and cost-effective technology for accurate diagnosis and treatment of TB. To address these limitations in this study, we have designed a multicenter validation roadmap we plan to carry out a phased multicenter validation roadmap in future. Phase 1 will engage TB consortium networks and pediatric TB centers across high-, intermediate-, and low-burden regions to recruit ≥1,000 prospectively enrolled patients (adults, children, HIV-positive, and immunocompromised). Standardized SOPs—including identical DNA extraction kits, spike-in controls (ERCC), and ≥20 M reads per sample-will be distributed to minimize inter-site variability. And, we will perform longitudinal sub-studies to examine whether the ≥0.05 RPM threshold remains valid. Phase 2 will test threshold portability on alternative platforms (Ion Torrent, Nanopore) and at reduced sequencing depths (5–10 M reads) typical of resource-limited laboratories. Phase 3 will integrate decision-analytic modeling to compare mNGS-guided versus standard diagnostic algorithms in terms of cost per accurate diagnosis, turnaround time, and patient outcomes. Meanwhile, RPM dynamics were incorporated into the health economics model to evaluate the cost-effectiveness of personalized treatment based on RPM in reducing toxic and side effects and QALY.

## Data Availability

Metagenomic sequencing data without reads of human genome was publicly available in NCBI with the SRA accession PRJNA1052360.
