# Supplementary figures and images for: Optimization of decision thresholds for Mycobacterium tuberculosis can effectively improve the performance of mNGS in tuberculosis diagnosis
Source: Front Cell Infect Microbiol. 2025 Sep 11;15:1646194. doi: 10.3389/fcimb.2025.1646194 (PMC12485630; doi:10.3389/fcimb.2025.1646194)

# Clinical utility of mNGS probability (RPKM calibrated)

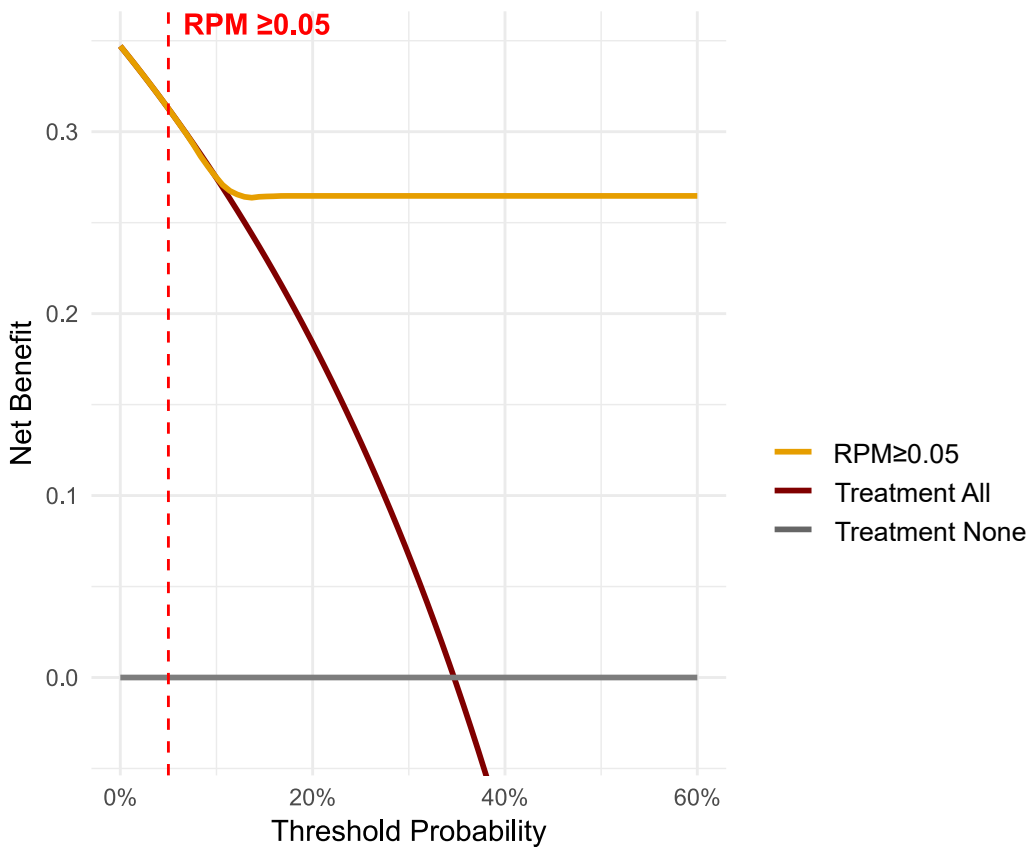

Supplement: Supplementary file 1 [file DataSheet1.pdf]
