# Supplementary material for: Optimization of decision thresholds for Mycobacterium tuberculosis can effectively improve the performance of mNGS in tuberculosis diagnosis
Source: Front Cell Infect Microbiol. 2025 Sep 11;15:1646194. doi: 10.3389/fcimb.2025.1646194 (PMC12485630; doi:10.3389/fcimb.2025.1646194)
Supplement: Supplementary file 2 [file DataSheet2.pdf]

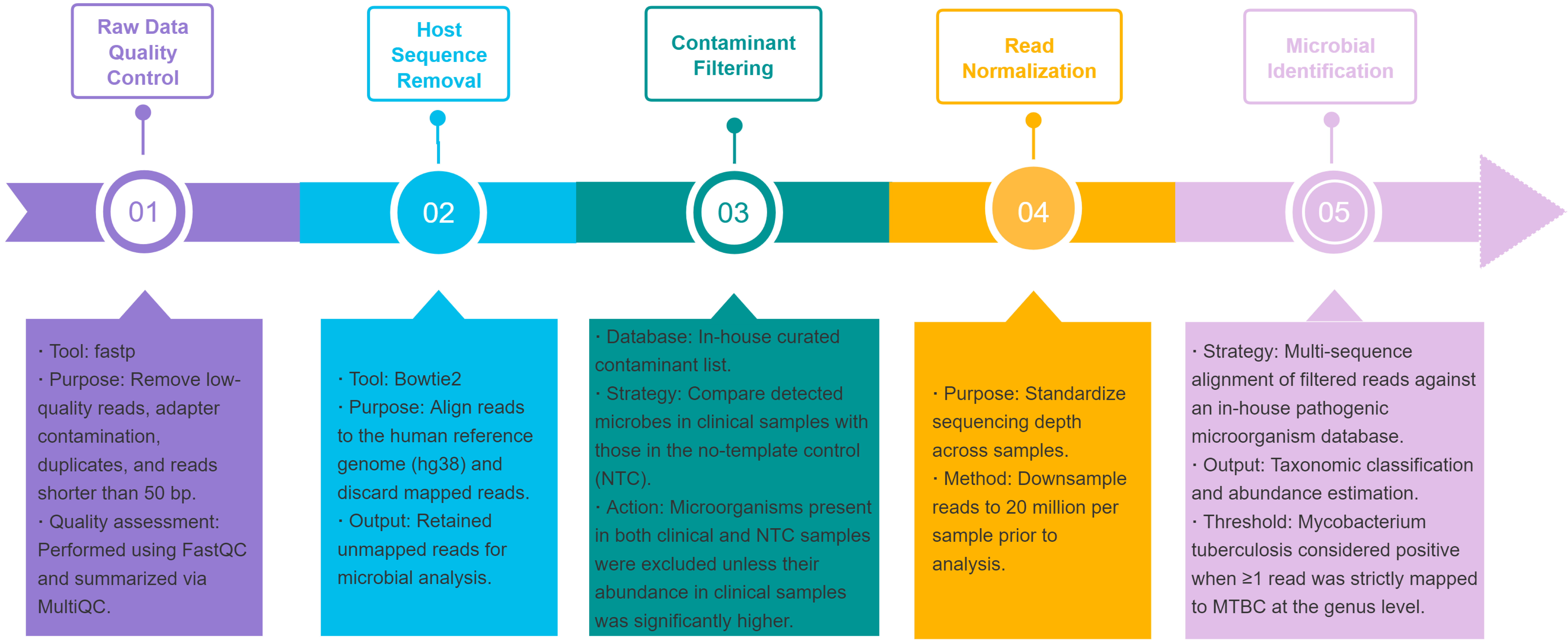
Raw Data Quality Control
01
· Tool: fastp
· Purpose: Remove low-quality reads, adapter contamination, duplicates, and reads shorter than 50 bp.
· Quality assessment: Performed using FastQC and summarized via MultiQC.
Host Sequence Removal
02
· Tool: Bowtie2
· Purpose: Align reads to the human reference genome (hg38) and discard mapped reads.
· Output: Retained unmapped reads for microbial analysis.
Contaminant Filtering
03
· Database: In-house curated contaminant list.
· Strategy: Compare detected microbes in clinical samples with those in the no-template control (NTC).
· Action: Microorganisms present in both clinical and NTC samples were excluded unless their abundance in clinical samples was significantly higher.
Read Normalization
04
· Purpose: Standardize sequencing depth across samples.
· Method: Downsample reads to 20 million per sample prior to analysis.
Microbial Identification
05
· Strategy: Multi-sequence alignment of filtered reads against an in-house pathogenic microorganism database.
· Output: Taxonomic classification and abundance estimation.
· Threshold: Mycobacterium tuberculosis considered positive when ≥1 read was strictly mapped to MTBC at the genus level.
